# The Epidemiology and Predictors of Outcomes Among Confirmed COVID-19 Cases in a Large Community Healthcare System in South Florida

**DOI:** 10.1007/s10900-020-00957-y

**Published:** 2021-01-07

**Authors:** Shenae Samuels, Jianli Niu, Candice Sareli, Paula Eckardt

**Affiliations:** 1 Office of Human Research, Memorial Healthcare System, 4411 Sheridan Street, Hollywood, FL 33021 USA; 2 Division of Infectious Disease, Memorial Healthcare System, 5647 Hollywood Boulevard, Hollywood, FL 33021 USA

**Keywords:** Epidemiology, Infectious disease, SARS-CoV-2, Racial/ethnic disparities, Outcomes

## Abstract

The novel coronavirus disease 2019 (COVID-19) continues to be a major public health concern. The aim of this study was to describe the presenting characteristics, epidemiology and predictors of outcomes among confirmed COVID-19 cases seen at a large community healthcare system which serves the epicenter and diverse region of Florida. We conducted a retrospective analysis of individuals with lab-confirmed SARS-CoV-2 infection who were seen, from March 2, 2020 to May 31, 2020, at Memorial Healthcare System in South Florida. Data was extracted from a COVID-19 registry of patients with lab-confirmed SARS-CoV-2 infection. Univariate and backward stepwise multivariate logistic regression models were used to determine predictors of key study outcomes. There were a total of 1692 confirmed COVID-19 patients included in this study. Increasing age was found to be a significant predictor of hospitalization, 30-day readmission and death. Having a temperature of 38 °C or more and increasing comorbidity score were also associated with an increased risk of hospitalization. Significant predictors of ICU admission included having a saturated oxygen level less than 90%, hypertension, dementia, rheumatologic disease, having a respiratory rate greater than 24 breaths per minute. Being of Hispanic ethnicity and immunosuppressant utilization greatly increased the risk of 30-day readmission. Having an oxygen saturation less than 90% and an underlying neurological disorder were associated with an increased likelihood of death. Results show that a patient’s demographic, underlying condition and vitals at triage may increase or reduce their risk of hospitalization, ICU admission, 30-day readmission or death.

**Supplementary Information:**

The online version contains supplementary material available at 10.1007/s10900-020-00957-y.

## Introduction

The 2019 novel coronavirus, caused by the Severe Acute Respiratory Syndrome Coronavirus -2 (SARS-CoV-2), has become one of the worst infectious disease outbreaks of recent times. In late December 2019, a cluster of patients were hospitalized with an initial diagnosis of pneumonia of unknown etiology in Wuhan, China [1]. The first confirmed case of SARS-CoV-2 in the United States (US) was reported on January 31, 2020 in Washington State [2]. On February 12, 2020, the World Health Organization (WHO) officially named the disease, caused by SARS-CoV-2, coronavirus disease 2019 (COVID-19) [3]. By March 11, 2020, WHO declared COVID-19 a worldwide pandemic [4]. Currently, the United States (US) has become the epicenter of the world with over 14 million confirmed cases and over 275,000 COVID-19 related deaths [5]. Within Florida, South Florida has become the epicenter of the state with over 400,000 confirmed cases and over 7000 deaths among Florida residents in the South Florida tri-county region of Miami-Dade, Broward and Palm Beach counties (data as of December 4, 2020) [6].

Although previous studies from China [7, 8] Italy [9], and most recently in the United States [10–12] have described the epidemiology of COVID-19 and the clinical features of disease, limited reports exist on the epidemiology of COVID-19 in the South Florida region, which comprises a high proportion of elderly residents that may create unprecedented health care challenges during the pandemic due to a higher prevalence of underlying health conditions and as such, their increased risk for severe COVID-19 disease. In March 2020, Memorial Healthcare System (MHS) together with the National Guard and the governor of Florida, opened a COVID-19 drive-through testing site. At the time of this analysis, there were 2415 cases tested at MHS drive-through sites. However, the focus of this study are confirmed COVID-19 cases who were seen and/or hospitalized at MHS facilities. This study examined the epidemiology, characteristics, and outcomes of individuals with confirmed SARS-CoV-2 infection at a large community-based health system in the South Florida region, with the aim of identifying risk factors for hospitalization, Intensive Care Unit (ICU) admission, 30-day readmission, and death. As an exploratory analysis, this study also examined admission status, discharge features, outcomes, as well as case-fatality rates by race/ethnicity to determine if racial/ethnic disparities exist within the community served by MHS.

## Methods

### Study Participants and Setting

The study’s population included patients who were seen during the first surge of COVID-19 cases at MHS facilities, from March 2, 2020 to May 31, 2020. MHS, which serves the South Florida community, is the third largest public healthcare system in the nation; consisting of five community hospitals, a freestanding children’s hospital and children’s specialty care center, primary care centers, urgent care centers, a nursing home, and a freestanding 24/7 care center.

### Data Sources

Patients with confirmed SARS-CoV-2 infection were based on a positive result of real time polymerase chain reaction (RT-PCR) assay of nasopharyngeal swab specimens. Data of patients with confirmed SARS-CoV-2 was extracted from MHS’ electronic medical records (EMR) and entered into REDCap, a secure web-based database, to develop a registry of lab-confirmed COVID-19 cases seen within MHS facilities and drive-through sites. This study was approved by MHS institutional review board (IRB # 2020.078).

Data points of this study included patient demographics, exposure characteristics, underlying disease/co-morbidities on record at time of initial testing, presenting vitals, home medications, signs and symptoms, and patient outcomes. Body mass index (BMI) was calculated as weight in kilograms divided by height in meters squared. In accordance with the Centers for Disease Control and Prevention (CDC), obesity was defined as having a BMI equal to or greater than 30.0 for adults (20 years and older) [13]. For compliance with Health Insurance Portability and Accountability Act (HIPAA) privacy requirements, all ages over 89 years old were coded as 90 years old. Age was further categorized into three groups: pediatric (0–17 years old), non-elderly (18–64 years old) and elderly (65 years and older).

### Outcome Measures

We assessed four key study outcomes: hospitalization, ICU admission, 30-day readmission and mortality. In the exploratory analysis of racial/ethnic disparities, other patient outcomes examined include hospital and ICU length of stay (LOS), the need for oxygen therapy or dialysis/renal treatment post discharge and case-fatality rates. For patients with multiple visits during the study’s period, the most severe outcome was assigned. For example, patients who were not admitted at initial visit, but were later admitted were assigned to non-ICU or ICU admission group, accordingly.

### Statistical Analysis

Descriptive statistics were calculated for all demographic and clinical characteristics. For comparisons of continuous variables, *t* test or analysis of variance (ANOVA) was used. Pearson’s Chi-Square test or its non-parametric equivalent was used for comparisons of categorical variables. As a part of the study’s exploratory analysis on racial/ethnic disparities, case-fatality rates by race/ethnicity groups was calculated as the number of deaths divided by the overall number of confirmed cases within that racial/ethnic group. Comorbidity score was calculated as the row total of the number of underlying conditions on record for each patient, which ranged from 0 to 9 in our study’s population. Comorbidity score was further categorized into four groups: individuals without underlying conditions; individuals with one underlying condition on record; individuals with two underlying conditions; and individuals with three or more underlying conditions on record. Symptomatic categories were also created by, first, calculating the row total for the number of symptoms on record for each patient (ranging from 0 to 10) followed by categorizing individuals with zero symptoms on record as “asymptomatic” and those with one or more symptoms on record as “symptomatic.”

To answer the study’s primary research question of identifying significant predictors of hospitalization, ICU admission, 30-day readmission, and death, univariate and backward stepwise multivariate logistic regression models were used. In univariate models, the following variables were examined against the outcome measures under study: demographics, presenting clinical characteristics, and home medications. Demographic variables included age in its continuous form, sex, race/ethnicity (non-Hispanic White, non-Hispanic Black or Hispanic), and smoking status (never smoker, current smoker or former smoker). Presenting clinical characteristics included seasonal flu vaccine status (yes or no), a history of being seen in the past 7 days for symptoms (yes or no), symptomatic category (symptomatic or asymptomatic), transfer status from another facility (yes or no), comorbidity score (0, 1, 2, or ≥ 3), the presence of individual underlying conditions (yes or no), and presenting vitals in their categorical forms such as saturated oxygen < 90% (yes or no), temperature ≥ 38.0 °C (yes or no) and respiratory rate > 24 breaths/min. (yes or no). Home medications, such as angiotensin-converting enzyme (ACE) inhibitors (yes or no), angiotensin II receptor blockers (ARBs) (yes or no), spironolactone (yes or no) and immunosuppressant (yes or no) were also included in univariate models.

Variables from univariate models with *P* < 0.10 were included in a backward stepwise logistic regression model. Variables with *P* < 0.05, from backward stepwise logistic regression models, were considered statistically significant predictors of the outcome measures under study. All analyses were performed using Stata/SE 15.1 statistical software.

## Results

### Demographics and Characteristics of Study Population

Demographic and clinical characteristics stratified by admission status are presented in Table 1. There were a total of 1692 confirmed cases of COVID-19 during the study period. Of cases with known admission status (n = 1537), a majority (67.9%) did not require hospital admission while 22.5% were hospitalized and 9.6% were admitted to the ICU. Among those not requiring hospital admission, over 8% died while almost 6% of those with a non-ICU admission died. Among the three admission types, ICU admissions had the highest proportion of deaths (48.8%). The average age of confirmed cases was 51 years old with the oldest mean age (65 years old) among patients who were admitted to the ICU. Among our study population, individuals with non-ICU admission were most likely to be female (51.6%), non-Hispanic (NH) Black (45.0%), non-elderly (51.9%) and have three or more underlying health conditions (43.4%). Patients admitted to the ICU were more likely to be male (61.6%), Hispanic (44.8%), elderly (57.5%) and with three or more underlying conditions (51.7%). Overall, majority of cases had no known travel exposure (91.8%) while over half of confirmed cases had a known close contact exposure (51.1%). Across all admission types, heart disease, hypertension, obesity, and diabetes were among the top five underlying conditions. In regards to cardiovascular home medications, ICU admissions had the highest proportion of individuals who were taking ACE inhibitors (18.2%) and ARBs (17.3%).

**Table 1:** Demographics and clinical characteristics of confirmed COVID-19 patients stratified by admission status (N = 1692)

	No admission (%)	Non-ICU admission (%)	ICU admission (%)	N (%)
Demographics				
N	1044 (67.9)	346 (22.5)	147 (9.6)	1537 (100.0)
Gender^†^				
Male	468 (45.0)	166 (48.4)	90 (61.6)	724 (47.4)
Female	571 (55.0)	177 (51.6)	56 (38.4)	804 (52.6)
Race/ethnicity*				
Non-Hispanic White	116 (12.9)	59 (18.4)	28 (22.4)	203 (15.1)
Non-Hispanic Black	400 (44.5)	144 (45.0)	41 (32.8)	585 (43.6)
Hispanic	382 (42.5)	117 (36.6)	56 (44.8)	555 (41.3)
Age (years), mean ± SD^†^	44.8 ± 16.8	62.0 ± 19.1	65.0 ± 16.1	51.0 ± 19.2
Age categories^†^				
Pediatric (0–17 years old)	37 (3.6)	3 (0.9)	2(1.4)	42 (2.8)
Non-elderly (18–64 years old)	882 (85.8)	177 (51.9)	60 (41.1)	1119 (73.9)
Elderly (65 years old and older)	109 (10.6)	161 (47.2)	84 (57.5)	354 (23.4)
Healthcare worker^†^				
Yes	154 (18.8)	19 (6.9)	3 (2.4)	176 (14.4)
No	667 (81.2)	258 (93.1)	120 (97.6)	1045 (85.6)
Known travel exposure				
Yes	79 (9.0)	19 (6.9)	7 (5.7)	105 (8.2)
No	797 (91.0)	258 (93.1)	116 (94.3)	1171 (91.8)
Known close contact exposure^†^				
Yes	472 (55.5)	98 (41.0)	42 (38.5)	612 (51.1)
No	378 (44.5)	141 (59.0)	67 (61.5)	586 (48.9)
Transfer from other facility^†^				
Yes	5 (0.5)	39 (11.4)	17 (11.6)	61 (4.1)
No	987 (99.5)	303 (88.6)	129 (88.4)	1419 (95.9)
Smoking status^†^				
Never	820 (86.0)	254 (78.2)	101 (73.2)	1175 (83.0)
Current	55 (5.8)	11 (3.4)	6 (4.4)	72 (5.1)
Former	79 (8.3)	60 (18.5)	31 (22.5)	170 (12.0)
Clinical characteristics				
Flu vaccine (2019–2020 season)				
Yes	95 (25.8)	27 (17.9)	13 (22.0)	135 (23.3)
No	274 (74.3)	124 (82.1)	46 (78.0)	444 (76.7)
Comorbidity score^†^				
0	301 (28.8)	29 (8.4)	12 (8.2)	342 (22.3)
1	384 (36.8)	75 (21.7)	26 (17.7)	485 (31.6)
2	214 (20.5)	92 (26.6)	33 (22.5)	339 (22.1)
≥ 3	145 (13.9)	150 (43.4)	76 (51.7)	371 (24.1)
Blood type				
A	47 (27.5)	36 (29.8)	22 (29.3)	105 (28.6)
B	35 (20.5)	20 (16.5)	20 (26.7)	75 (20.4)
AB	8 (5.3)	4 (3.3)	1 (1.3)	14 (3.8)
O	80 (46.8)	61 (50.4)	32 (42.7)	173 (47.1)
Heart disease^†^	53 (5.8)	75 (23.5)	33 (24.3)	161 (11.7)
Hypertension^†^	288 (30.4)	209 (63.5)	109 (75.7)	606 (42.7)
Obesity*	552 (56.0)	164 (47.8)	73 (50.3)	789 (53.5)
Pulmonary disease^†^	14 (1.5)	25 (7.9)	13 (9.6)	52 (3.8)
Diabetes with/without complications^†^	129 (12.4)	113 (32.7)	60 (40.8)	302 (19.7)
Asthma	101 (11.0)	32 (10.0)	10 (7.6)	143 (10.4)
Kidney disease^†^	18 (2.0)	37 (11.6)	19 (14.4)	74 (5.4)
Rheumatologic disease^†^	18 (2.0)	25 (8.0)	4 (3.0)	47 (3.5)
Liver disease	6 (0.7)	3 (1.0)	3 (2.3)	12 (0.9)
Hypothyroidism^†^	34 (3.7)	33 (10.5)	17 (13.3)	84 (6.2)
Hyperthyroidism	5 (0.6)	2 (0.7)	1 (0.8)	8 (0.6)
Dementia^†^	8 (0.9)	52 (16.5)	10 (7.6)	70 (5.2)
Currently pregnant	22 (5.6)	8 (6.2)	3 (7.3)	33 (5.8)
Post-partum*	2 (0.4)	5 (3.0)	0 (0.0)	7 (1.0)
Malnutrition*	1 (0.1)	5 (1.6)	2 (1.6)	8 (0.6)
Neurological disorder^†^	18 (2.0)	21 (6.6)	14 (10.8)	53 (3.9)
History of cancer diagnosis^†^	36 (3.9)	24 (7.8)	15 (11.5)	75 (5.6)
Current	10 (28.6)	9 (37.5)	4 (26.7)	23 (31.1)
Past	25 (71.4)	15 (62.5)	11 (73.3)	51 (68.9)
Hematologic disease*	19 (2.1)	11 (3.6)	8 (6.1)	38 (2.8)
Transplantation history	2 (0.2)	1 (0.3)	1 (0.8)	4 (0.3)
Kidney transplant	2 (100.0)	1 (100.0)	1 (100.0)	4 (100.00)
Immunosuppressant use	1 (50.0)	1 (100.0)	1 (100.0)	3 (75.0)
HIV/AIDS	5 (0.6)	2 (0.8)	0 (0.0)	7 (0.6)
HIV+ and currently on antiviral therapy (ART)	4 (80.00)	2 (100.0)	0 (0.0)	6 (85.7)
Angiotensin-converting enzyme (ACE) inhibitors^†^	55 (5.9)	42 (13.0)	24 (18.2)	121 (8.7)
Angiotensin II receptor blockers (ARBs)^†^	47 (5.0)	48 (14.7)	23 (17.3)	118 (8.4)
Mortality status^†^				
Alive	63 (91.3)	303 (94.1)	64 (51.2)	430 (83.3)
Death	6 (8.7)	19 (5.9)	61 (48.8)	86 (16.7)

^*^P < 0.05; ^†^P ≤ 0.001

Percentages may not add to 100% due to rounding error and missing data

Categorical variables were calculated using Pearson’s chi-square or Fisher’s exact test where appropriate and are presented as frequencies and proportions

Continuous variables were calculated using one-way ANOVA and are presented as mean ± standard deviation (SD)

Table 2 depicts that, compared to individuals not requiring admission, a greater proportion of individuals requiring general admission and admission to the ICU had temperatures ≥ 38 °C (febrile state), heart rates ≥ 100 beats per minute (tachycardia), respiratory rates > 24 breaths per minute (tachypnea), and saturated oxygen < 90% (hypoxemia). Across all admission types, the five most common symptoms were fever, cough, myalgia, malaise and shortness of breath.

**Table 2:** Presenting vitals, and signs and symptoms of confirmed COVID-19 patients stratified by admission status (N = 1692)

	No Admission (%)	Non-ICU Admission (%)	ICU Admission (%)	N (%)
Presenting vitals				
Temperature ≥ 38.0 °C^†^	266 (25.5)	139 (40.2)	69 (46.9)	474 (30.8)
Heart rate ≥ 100 beats/min	412 (39.5)	143 (41.3)	66 (44.9)	621 (40.4)
Respiratory rate > 24 breaths/min^†^	73 (7.0)	47 (13.6)	44 (29.9)	164 (10.7)
Saturated oxygen < 90%^†^	6 (0.6)	29 (8.4)	36 (24.5)	71 (4.6)
Signs and symptoms				
History of being seen in the past 7 days for symptoms^†^	150 (16.7)	75 (24.0)	36 (27.3)	261 (19.5)
Symptomatic category*				
Asymptomatic	76 (7.3)	25 (7.2)	2 (1.4)	103 (6.7)
≥ 1 symptoms	968 (92.7)	321 (92.8)	145 (98.6)	1434 (93.3)
Influenza infection	12 (1.9)	2 (0.9)	5 (4.2)	19 (1.9)
Fever^†^	594 (58.2)	230 (69.3)	104 (73.8)	928 (62.2)
Cough	708 (69.3)	212 (65.2)	101 (74.3)	1021 (68.9)
Non-productive cough	620 (87.6)	169 (79.7)	83 (82.2)	872 (85.4)
Cough with sputum	46 (6.5)	37 (17.5)	15 (14.9)	98 (9.6)
Cough with bloody sputum	6 (0.9)	3 (1.4)	1 (1.0)	10 (1.0)
Loss of taste and/or smell	51 (9.2)	11 (7.8)	4 (8.5)	66 (8.9)
Sore throat^†^	175 (17.7)	25 (8.9)	16 (13.2)	216 (15.5)
Rhinorrhea	84 (8.8)	18 (6.5)	6 (4.8)	108 (8.0)
Ear pain	15 (1.6)	2 (0.8)	2 (1.8)	19 (1.4)
Wheezing^†^	25 (2.5)	16 (5.0)	11 (8.1)	52 (3.6)
Chest pain	91 (9.1)	38 (12.1)	12 (8.8)	141 (9.7)
Myalgia	323 (33.3)	86 (29.2)	30 (24.4)	439 (31.6)
Arthralgia	20 (2.1)	6 (2.1)	2 (1.6)	28 (2.1)
Malaise^†^	194 (20.4)	88 (30.7)	43 (34.1)	325 (23.8)
Shortness of breath^†^	227 (22.6)	162 (50.9)	81 (58.7)	470 (32.2)
Headache^†^	172 (17.6)	26 (8.8)	21 (16.4)	219 (15.7)
Altered consciousness^†^	4 (0.4)	39 (12.0)	17 (12.0)	60 (4.1)
Seizure	3 (0.3)	3 (1.1)	0 (0.0)	6 (0.5)
Abdominal pain^†^	37 (3.7)	36 (11.3)	8 (6.2)	81 (5.6)
Vomiting/nausea*	95 (9.4)	49 (15.5)	20 (14.7)	164 (11.2)
Diarrhea^†^	113 (11.4)	69 (21.8)	25 (18.9)	207 (14.4)
Conjunctivitis	4 (0.4)	0 (0.0)	2 (1.5)	6 (0.4)
Lymphadenopathy	3 (0.3)	2 (0.7)	0 (0.0)	5 (0.4)
Bleeding/hemorrhage	5 (0.5)	4 (1.4)	1 (0.8)	10 (0.7)
Clinical pneumonia^†^	81 (9.0)	181 (56.9)	96 (70.6)	358 (26.5)

*P < 0.05

^†^P ≤ 0.001

Percentages may not add to 100% due to rounding error and missing data

Categorical variables were calculated using Pearson’s chi-square or Fisher’s exact test where appropriate and are presented as frequencies and proportions

Continuous variables were calculated using one-way ANOVA and are presented as mean ± standard deviation (SD)

### Predictors of Hospitalization, ICU Admission, 30-Day Readmission and Death

Tables 3 presents results of the study’s main analyses assessing predictors of key study outcomes. Increasing age and febrile state were found to be significant predictors of hospitalization. Having one or more underlying condition was also found to be a significant predictor of hospitalization; of which, having 3 or more underlying conditions had the strongest association with hospitalization (OR = 7.67; *P* = 0.000).

**Table 3:** Multivariate (backward selection) logistic analysis of predictors of hospitalization, ICU admission, 30-day readmission and death

	OR (95% CI)	P value
Hospitalization^a^		
Age	1.04 (1.02, 1.05)	0.000
Temperature ≥ 38.0 °C	2.13 (1.23, 3.70)	0.007
Comorbidity score		
1	4.10 (1.65, 10.2)	0.002
2	3.41 (1.33, 8.76)	0.011
≥ 3	7.67 (2.98, 19.76)	0.000
ICU admission^b^		
Dementia	0.43 (0.20, 0.92)	0.030
Rheumatologic disease	0.14 (0.03, 0.65)	0.012
Saturated oxygen < 90%	2.84 (1.52, 5.31)	0.001
Hypertension	2.17 (1.34, 3.53)	0.002
Respiratory rate > 24 breaths/min	2.54 (1.48, 4.34)	0.001
30-day readmission^c^		
Age	1.05 (1.01, 1.09)	0.010
Hispanic ethnicity	7.23 (1.42, 36.9)	0.017
Immunosuppressant	9.57 (1.19, 77.0)	0.034
Death^d^		
Age	1.08 (1.05, 1.11)	0.000
Saturated oxygen < 90%	5.89 (2.39, 14.5)	0.000
Neurological disorder	4.60 (1.39, 15.26)	0.013

*CI* confidence interval

^a^Variables with *P* < 0.10 included in the multivariable model are as follows: age, sex, race/ethnicity (NH White vs. NH Black vs. Hispanic), smoking status, flu vaccine status, comorbidity score (0 vs. 1 vs. 2 vs. ≥ 3), chronic cardiac disease, hypertension, obesity status, diabetes with/without complications, kidney disease, rheumatologic disease, hypothyroidism, dementia, malignant neoplasm, hematologic disease, angiotensin-converting enzyme (ACE) inhibitors, angiotensin II receptor blockers (ARBs), spironolactone, immunosuppressant, history of being seen in the past 7 days for symptoms, respiratory rate > 24 breaths/min., and temperature ≥ 38.0 °C

^b^Variables with *P* < 0.10 included in the multivariable model are as follows: age, sex, comorbidity score (0 vs. 1 vs. 2 vs. ≥ 3), hypertension, diabetes with/without complications, rheumatologic disease, dementia, neurological disorder, angiotensin-converting enzyme (ACE) inhibitors, symptomatic category (asymptomatic vs. symptomatic), respiratory rate > 24 breaths/min., and saturated oxygen < 90%

^c^Variables with *P* < 0.10 included in the multivariable model are as follows: age, race/ethnicity (NH White vs. NH Black vs. Hispanic), hypertension, pulmonary disease, clinical pneumonia, angiotensin-converting enzyme (ACE) inhibitors, and immunosuppressant

^d^Variables with *P* < 0.10 included in the multivariable model are as follows: age, transfer status, sex, race/ethnicity (NH White vs. NH Black vs. Hispanic), smoking status, comorbidity score (0 vs. 1 vs. 2 vs. ≥ 3), chronic cardiac disease, hypertension, pulmonary disease, kidney disease, liver disease, hypothyroidism, dementia, neurological disorder, malignant neoplasm, hematologic disease, respiratory rate > 24 breaths/min., and saturated oxygen < 90%

In terms of ICU admission, tachypnea, hypoxemia and hypertension were all associated with an increased likelihood of ICU admission. However, having a pre-existing rheumatologic disease (OR = 0.14; *P* = 0.012) or dementia (OR = 0.43; *P* = 0.030) was associated with a reduced likelihood of being admitted to the ICU.

Increasing age was a significant predictor of both 30-day readmission and death. Compared to NH Whites, being of Hispanic ethnicity was associated with an increased likelihood of being readmitted within 30 days of discharge (OR = 7.23; *P* = 0.017). Immunosuppressant use was also found to be a significant predictor of 30-day readmission (OR = 9.57; *P* = 0.034). However, the rarity of immunosuppressant use may have contributed to the wide 95% confidence interval (CI) in the multivariate logistic regression model. Consequently, our study’s estimate may not be a precise measure of the true association between immunosuppressant use and 30-day readmission. In addition to increasing age, hypoxemia (OR = 5.89; *P* = 0.000) and having a neurological disorder (OR = 4.60; *P* = 0.013) were both significant predictors of an increased likelihood of death.

### Exploratory Analysis: Racial/Ethnic Disparities Among COVID-19 Patients

An exploratory analysis was conducted to determine if racial/ethnic disparities exist in our study population. Table 4 shows significant differences in admission status, hospital LOS, and mortality status by race/ethnicity groups. The proportion of NH Blacks and Hispanic COVID-19 cases was more than double that of NH Whites (43.5%, 41.6% and 14.9%, respectively). Consequently, NH Blacks and Hispanics accounted for larger proportions of all admission types (P = 0.003). Among race/ethnicity groups, Hispanics had the longest average hospital LOS (P = 0.008). While NH Whites accounted for the lowest proportion of confirmed COVID-19 cases and admissions, results show that NH Whites accounted for the highest proportion of deaths (P = 0.000). Case-fatality rate was highest for NH Whites (14.0%) followed by Hispanics (4.1%) and non-Hispanic Blacks (3.3%).

**Table 4:** Admission Status, Discharge Features, Outcomes and Case-Fatality Rates by Race/Ethnicity

	Non-Hispanic White (%)	Non-Hispanic Black (%)	Hispanic (%)	P value
N	221 (14.9)	643 (43.5)	615 (41.6)	–
Admission status				**0.003**
No admission	116 (12.9)	400 (44.5)	382 (42.5)	
Non-ICU admission	59 (18.4)	144 (45.0)	117 (36.6)	
ICU admission	28 (22.4)	41 (32.8)	56 (44.8)	
Discharge treatment				
Post-discharge oxygen therapy				0.070
Yes	21 (25.9)	31 (38.3)	29 (35.8)	
No	56 (15.3)	166 (45.4)	144 (39.3)	
Post-discharge dialysis/renal treatment				0.842
Yes	4 (18.2)	11 (50.0)	7 (31.8)	
No	75 (17.9)	183 (43.6)	162 (38.6)	
Outcomes				
Length of hospitalization in days, mean ± SD	14.0 ± 14.0	10.9 ± 12.7	15.3 ± 17.7	**0.008**
Length of ICU stay in days, mean ± SD	12.2 ± 13.5	16.7 ± 13.9	18.4 ± 17.9	0.232
Mortality status				**0.000**
Alive	64 (16.0)	181 (45.1)	156 (38.9)	
Death	31 (40.3)	21 (27.3)	25 (32.5)	
Case-fatality rate (%)^a^	14.0	3.3	4.1	

Bold font indicates statistical significance at *P* < 0.05

*SD* standard deviation

Percentages may not add to 100% due to rounding error and missing data

Categorical variables are presented as frequency and proportion

Continuous variables are presented as mean ± SD or median (range)

^a^Case-fatality rate calculated as the number of deaths divided by the overall number of confirmed cases

## Discussion

Given the novelty of SARS-CoV-2, a paucity of report exists to describe the presenting characteristics, epidemiology and predictors of outcomes among confirmed cases of COVID-19 in the US. In this study, we present characteristics of individuals with laboratory confirmed COVID-19 in South Florida. Of 1537 confirmed COVID-19 cases with known admission status data, majority of patients did not require hospital admission while 22.5% required hospital admission, 9.6% required intensive care, and over 16% of patients died. This finding is in line with other studies that have shown that a majority of COVID-19 patients have mild disease [14]. In our study population, the proportion of individuals requiring intensive care and death rates were slightly lower than that of a study conducted in the New York City region which found that of the hospitalized patients, 14.2% were admitted to the ICU, and 24.5% died [12].

The New York City area study reported hypertension, obesity, and diabetes among the most common comorbidities [12]. Further, a report of 1482 patients admitted during March 1–30, 2020 in 14 US states found that among patients with data on underlying conditions, almost 90% of patients had one or more underlying conditions; of which, hypertension, obesity, chronic lung disease, diabetes mellitus, and cardiovascular disease were the most common [10]. Similarly, obesity, hypertension and diabetes were among the most prevalent underlying conditions in our study population and over 77% of our study population had one or more underlying condition. Similar to previous studies, majority of confirmed COVID-19 patients were symptomatic with the most common presenting signs and symptoms including cough, fever, myalgia, malaise, and shortness of breath [8–12, 14–16]. Exposure by close contact occurred more frequently than exposure by travel suggesting that community spread fueled COVID-19 rates within the South Florida region. The CDC has developed separate public health guidelines for community-related exposure versus travel-related exposure. Our results would suggest the need for continued emphasis on mitigating transmission through social distancing and personal prevention, such as hand-washing and wearing of a mask while in the presence of people outside of one’s household, as well as adhering to the CDC-recommended guidance for known or suspected exposure to a person with COVID-19 [17].

Several demographics and clinical characteristics that associate with increased risk of severity of COVID-19 requiring hospitalization and/or intensive care in people infected with SARS-CoV-2, have been proposed [11, 18]. Among the factors identified as increasing risk of severe outcomes, racial/ethnic minorities have been reported to be severely or disproportionately impacted [11, 19–21]. While our study did not show that race/ethnicity was independently associated with severe COVID-19 outcomes (ICU admission and death), Hispanic ethnicity was found to be associated with an increased risk of 30-day readmission. It is well known that patients without a personal doctor are more likely to utilize the emergency department (ED) as their usual source of care. It is possible that patients without a personal doctor, and consequently poor post-discharge support (such as the inability to complete follow-up care with a primary care provider), may be more likely to present to the ED for acute care and require readmission compared to those with a personal doctor. In our service community of Broward and Miami-Dade counties, the Hispanic population account for the highest proportion of individuals without a personal doctor [22], which may play a role in the observed increased risk of 30-day readmission. In addition, compared to non-Hispanics, Hispanics living in Florida are more likely to live in multigenerational households which may present a challenge for COVID-19 spread and recovery [23]. Particularly, as additional evidence emerges to suggest the possibility COVID-19 reinfections [24–27]. Given the possibility of reinfection, it has been suggested that self-isolation after discharge may be an effective practice in reducing the risk of readmission [28].

Rheumatologic disease and dementia were found to be associated with a reduced risk of ICU admission. Research has suggested that some disease modifying anti-rheumatic drugs (DMARDS), taken by patients with rheumatologic disease, may decrease the cytokine storm associated with severe COVID-19 illness [29]. In our study population, patients with dementia may account for nursing home patients with mild disease who were admitted due to isolation inability at their respective nursing home facilities.

Our exploratory analysis showed that NH Blacks and Hispanics were disproportionately represented among both outpatient and inpatient COVID-19 patient groups. These observed racial/ethnic disparities may be, in part, due to an interplay of biomedical and socioeconomic factors. Blacks and Hispanics are more likely to live and work in environments that increase their risk for COVID-19 infection. Namely, studies have shown that 24% of Blacks and Hispanics work in the service industry compared to 16% of Whites [30]. Approximately 41% of Blacks and 38% of Hispanics report multiunit residential buildings surrounding their residences compared to 23% of Whites [31]. Generally, communities of color have a greater prevalence of underlying comorbidities that increase their risk for severe COVID-19.

However, although NH Blacks and Hispanics accounted for a larger proportion of COVID-19 cases and admissions, NH Whites in our study population were more likely to die from COVID-19. It is well documented that older age increases the risk of COVD-19 related death [7, 9, 32]. Almost 50% of NH Whites seen at our facilities were elderly compared to 16.4% of NH Blacks and 26.5% of Hispanics. Furthermore, a greater proportion of NH Whites (35.8%) had 3 or more underlying conditions compared to non-Hispanic Blacks (26.6%) and Hispanics (21.6%) (Table S1). As such, the older age distribution and higher prevalence of a co-morbidity score ≥ 3 may explain the higher death rates among NH Whites presenting to our facilities compared to NH Blacks and Hispanics.

While age showed strong associations with hospitalization, 30-day readmission and death, we did not find an increased risk for ICU admission. This may be explained by the fact that the elderly represented the greatest proportion of ICU admissions in our studied cohort which may be a confounder influencing these results.

The comorbidities we identified as associated with hospital admission of COVID-19 are largely similar to previous reports [10, 12]. Among confirmed COVID-19 cases, a higher comorbidity score was significantly associated with an increased risk of hospitalization. Furthermore, certain underlying health conditions, such as hypertension and neurological disorder, were associated with an increased risk of COVID-19 severe outcomes. Many of these underlying health conditions are common among elderly Florida residents [33]. Thus, our findings suggest that the COVID-19 surveillance and mitigation efforts should have an increased focus on the elderly and individuals with underlying health conditions.

## Conclusion

Our study indicates that the presence of comorbidities, particularly hypertension, and neurological disorders are independent predictors for severe COVID-19 infection requiring ICU admission and increased risk of death, respectively. Furthermore, increasing age and poor vitals at triage (hypoxemia, tachypnea, and febrile state) were associated with an increased risk of hospitalization, ICU admission and death. Among our study populations, increasing age and Hispanic ethnicity were associated with an increased risk of 30-day readmission. Early identification of these risk factors may help in clinical decision making and provide prompt treatment intervention for these high risk patients.

## Supplementary Information

Below is the link to the electronic supplementary material.

Supplementary file1 (DOCX 15 KB)

## References

[1] Rothan HA, Byrareddy SN (2020). The epidemiology and pathogenesis of coronavirus disease (COVID-19) outbreak. Journal of Autoimmunity.

[2] Holshue ML, DeBolt C, Lindquist S (2020). First case of 2019 novel coronavirus in the United States. New England Journal of Medicine.

[3] Jan H, Faisal S, Khan A (2020). COVID-19: Review of epidemiology and potential treatments against 2019 novel coronavirus. Discoveries.

[4] WHO. (2020) WHO Director-General’s opening remarks at the media briefing on COVID-19-11 March 2020. https://www.who.int/dg/speeches/detail/who-director-general-s-opening-remarks-at-the-media-briefing-on-covid-19---11-march-2020.

[5] CSSE. (2020) COVID-19 Dashboard by the Center for Systems Science and Engineering (CSSE) at Johns Hopkins University. https://coronavirus.jhu.edu/map.html.

[6] Florida’s COVID-19 Data and Surveillance Dashboard. https://experience.arcgis.com/experience/96dd742462124fa0b38ddedb9b25e429.

[7] Zhou F, Yu T, Du R (2020). Clinical course and risk factors for mortality of adult inpatients with COVID-19 in Wuhan, China: A retrospective cohort study. The Lancet.

[8] Yang W, Cao Q, Qin L (2020). Clinical characteristics and imaging manifestations of the 2019 novel coronavirus disease (COVID-19): A multi-center study in Wenzhou city, Zhejiang. China. Journal of Infection.

[9] Onder G, Rezza G, Brusaferro S (2020). Case-fatality rate and characteristics of patients dying in relation to COVID-19 in Italy. JAMA.

[10] Garg S (2020). Hospitalization rates and characteristics of patients hospitalized with laboratory-confirmed coronavirus disease 2019—COVID-NET, 14 States, March 1–30, 2020. MMWR Morbidity And Mortality Weekly Report.

[11] Azar KM, Shen Z, Romanelli RJ (2020). Disparities in outcomes among covid-19 patients in a large health care system. In California: Study examines disparities in access and outcomes for COVID-19 patients who are members of racial and ethnic minorities and socioeconomically disadvantaged groups. Health Affairs.

[12] Richardson S, Hirsch JS, Narasimhan M (2020). Presenting characteristics, comorbidities, and outcomes among 5700 patients hospitalized with COVID-19 in the New York City area. JAMA.

[13] CDC. (2020) About Adult BMI. https://www.cdc.gov/healthyweight/assessing/bmi/adult_bmi/index.html.

[14] Hassan SA, Sheikh FN, Jamal S (2020). Coronavirus (COVID-19): A review of clinical features, diagnosis, and treatment. Cureus.

[15] Chen H, Guo J, Wang C (2020). Clinical characteristics and intrauterine vertical transmission potential of COVID-19 infection in nine pregnant women: A retrospective review of medical records. The Lancet.

[16] Wang D, Hu B, Hu C (2020). Clinical characteristics of 138 hospitalized patients with 2019 novel coronavirus-infected pneumonia in Wuhan, China. JAMA.

[17] CDC. (2020). Public health guidance for community-related exposure. https://www.cdc.gov/coronavirus/2019-ncov/php/public-health-recommendations.html.

[18] Petrilli CM, Jones SA, Yang J (2020). Factors associated with hospital admission and critical illness among 5279 people with coronavirus disease 2019 in New York City: Prospective cohort study. BMJ.

[19] Yancy CW (2020). COVID-19 and African Americans. JAMA.

[20] Webb Hooper M, Nápoles AM, Pérez-Stable EJ (2020). COVID-19 and racial/ethnic disparities. JAMA.

[21] Artiga, S., Garfield, R., & Orgera, K. (2020). Communities of color at higher risk for health and economic challenges due to COVID-19. In The Henry J. Kaiser Family Foundation. Retrieved April 17, 2020, from https://www.kff.org/disparities-policy/issue-brief/communities-of-color-at-higher-risk-for-health-and-economic-challenges-due-to-covid-19/.

[22] FLHealthCHARTS - Population Estimates. Retrieved April 25, 2020, from http://www.flhealthcharts.com/flquery/population/populationrpt.aspx.

[23] Carrozza, M. (2020). Distribution of multigenerational households by race and ethnicity, and implications for COVID-19 planning. In *Geospatial research briefs*. https://www.healthlandscape.org/Geospatial-Brief-Multigenerational-Households.pdf.

[24] Tillett, R., Sevinsky, J., Hartley, P., et al. (2020). Genomic evidence for a case of reinfection with SARS-CoV-2. SSRN. https://ssrn.com/abstract=3680955. 10.1016/S1473-3099(20)30764-7 PMC7550103 33058797

[25] Prado-Vivar, B., Becerra-Wong, M., & Guadalupe, J. J., et al. (2020). COVID-19 Re-infection by a phylogenetically distinct SARS-CoV-2 variant. First Confirmed Event in South America September 3, 2020.

[26] To KK-W, Hung IF-N, Ip JD (2020). COVID-19 re-infection by a phylogenetically distinct SARS-coronavirus-2 strain confirmed by whole genome sequencing. Clinical Infectious Diseases.

[27] Van Elslande JJ, Vermeersch P, Vandervoort K (2020). Symptomatic SARS-CoV-2 reinfection by a phylogenetically distinct strain. Clinical Infectious Diseases: An Official Publication of the Infectious Diseases Society of America.

[28] Wang X, Xu H, Jiang H (2020). The clinical features and outcomes of discharged coronavirus disease 2019 patients: A prospective cohort study. QJM: An International Journal of Medicine.

[29] Kastritis E, Kitas GD, Vassilopoulos D (2020). Systemic autoimmune diseases, anti-rheumatic therapies, COVID-19 infection risk and patient outcomes. Rheumatology International.

[30] BLS. (2019). Report 1082: Labor force characteristics by race and ethnicity, 2018. Retrieved January 1, 2020, from https://www.bls.gov/opub/reports/race-and-ethnicity/2018/home.htm.

[31] KFF analysis of 2017 American Housing Survey data. https://www.census.gov/data/data-tools/ahs-table-creator.html.

[32] Mahase E (2020). Covid-19: Death rate is 0.66% and increases with age, study estimates. BMJ: British Medical Journal.

[33] Zevallos JC, Wilcox ML, Jean N, Acuna JM (2016). Profile of the older population living in Miami-Dade County, Florida: An observational study. Medicine.

