# Supplementary material for: The Epidemiology and Predictors of Outcomes Among Confirmed COVID-19 Cases in a Large Community Healthcare System in South Florida
Source: J Community Health. 2021 Jan 7;46(4):822–31. doi: 10.1007/s10900-020-00957-y (PMC7787879; doi:10.1007/s10900-020-00957-y)
Supplement: Supplementary file 1 — Supplementary file1 (DOCX 15 KB) [file 10900_2020_957_MOESM1_ESM.docx]

| Table S1. Select Demographics and Underlying Comorbidities by Race/Ethnicity | | | | |
| --- | --- | --- | --- | --- |
|  | **Non-Hispanic White (%)** | **Non-Hispanic Black (%)** | **Hispanic (%)** | **P-Value** |
| Median Age in Years (Range) | 62.8 (16.9-90) | 46.5 (0.9-87.9) | 52.9 (12.6-90) | - |
| Age Categories |  |  |  | **0.000** |
| Pediatric (0-17 years old) | 3 (1.4) | 26 (4.1) | 14 (2.3) |  |
| Non-Elderly (18-64 years old) | 113 (52.3) | 504 (79.5) | 430 (71.2) |  |
| Elderly (65 years old and older) | 100 (46.3) | 104 (16.4) | 160 (26.5) |  |
| Comorbidity Score |  |  |  | **0.000** |
| 0 | 43 (19.5) | 132 (20.5) | 154 (25.0) |  |
| 1 | 54 (24.4) | 180 (28.0) | 214 (34.8) |  |
| 2 | 45 (20.4) | 160 (24.9) | 114 (18.5) |  |
| ≥ 3 | 79 (35.8) | 171 (26.6) | 133 (21.6) |  |
| Neurological Disorder | 17 (8.5) | 20 (3.4) | 18 (3.3) | **0.003** |
| Chronic Cardiac Disease | 60 (29.4) | 52 (8.8) | 53 (9.6) | **0.000** |
| Hypertension | 98 (46.2) | 279 (45.5) | 231 (40.4) | 0.142 |
| Obesity | 76 (35.4) | 370 (60.9) | 287 (48.5) | **0.000** |
| Diabetes with/without complications | 40 (18.1) | 148 (23.0) | 115 (18.7) | 0.105 |
| Pulmonary Disease | 14 (6.9) | 16 (2.7) | 27 (4.9) | **0.023** |
| Asthma | 17 (8.5) | 60 (10.0) | 66 (12.0) | 0.329 |
| Immunosuppressant | 3 (2.1) | 5 (1.2) | 8 (2.1) | 0.551 |
| Bold font indicates statistical significance at *P* <0.05  SD= Standard Deviation  Percentages may not add to 100% due to rounding error and missing data  Categorical variables are presented as frequency and proportion  Continuous variables are presented as Mean ± SD or Median (Range) | | | | |
